# Summer Camp Clinical Placements in Young Families Nursing: An Interpretive Description Study

**DOI:** 10.1177/08445621241288489

**Published:** 2024-10-14

**Authors:** Claire Williams, Renée Gordon, Emily Richard

**Affiliations:** 1 Faculty of Nursing, 3427 University of New Brunswick, Moncton, NB, Canada

**Keywords:** Clinical nursing education, camp nursing, young families health, interpretative description, paediatric nursing clinical

## Abstract

**Purpose:**

Obtaining meaningful clinical experiences with paediatric and perinatal (young families) populations is increasingly challenging for nursing programs. Care for this population has largely moved to outpatient and tertiary settings. Therefore, a current trend is to use non-traditional clinical settings. While summer camps offer rich learning experiences for nursing students, they are seldom used as clinical placements. Faculty at an Atlantic Canadian university sought a novel way to engage students in young families’ learning by partnering with overnight summer camps, staffed by counsellors, camp administrators, and on-site nurses. Campers included those with lived experience of cancer, diabetes, physical and developmental challenges, and socioeconomic challenges. This study assesses how young families course outcomes were met by an innovative clinical experience within a Bachelor of Nursing program and describes the perceptions and experiences of those involved.

**Methods:**

Study participants included students (n = 4), camp directors (n = 3), a camp nurse (n = 1), and the clinical educator (n = 1). Data collection involved semi-structured interviews and a focus group. Interpretive description methodology was used to identify themes and patterns related to overarching research questions.

**Findings:**

Students met the outcomes and overall participant perceptions and experiences were positive. However, some participants shared constructive critiques for future consideration.

**Conclusions:**

Nursing students who completed a young families clinical placement at summer camps met course outcomes, and those involved reported both positive experiences and constructive critiques for future consideration.

Obtaining meaningful paediatric clinical experiences has been a longstanding challenge in nursing education (Evans, 2017; Hendrickx et al., 2020), exacerbated by increased enrolment in nursing education programs in response to the nursing workforce shortage (Canadian Association of Schools of Nursing [CASN], 2021).

Overnight summer camps for children and adolescents are a well-established and arguably ubiquitous, feature of summertime in Canada. Camp nurses play a key role in the health and wellbeing of campers and camp staff. Moreover, there is a wide range of summer camps available, including those that focus on children and youth from socially and economically excluded communities or those with specific health conditions such as diabetes, cancer, hemophilia, etc. Yet, summer camps are seldom used as clinical placements in nursing education to meet young families’ learning outcomes.

Given the important role that nurses play at summer camps and the rich learning experiences they can offer students, faculty at one Atlantic Canada university partnered with summer camps to provide nursing students with a unique placement option for their young families’ clinical course in the summer of 2022. This paper describes the innovative young families clinical experience, including how course outcomes were met, and an exploration of the perceptions and experiences of the students, faculty, and camp staff involved in this placement.

## Background

In Canada, baccalaureate nursing education is designed to prepare graduates for generalist nursing practice through the provision of broad-based programming, including foundational knowledge across the nursing spectrum and lifespan, rather than in-depth specialized knowledge in a particular specialty area (Canadian Council of Registered Nurse Regulators, 2018; CASN, 2016, 2022). As part of this curricular framework, nursing education programs typically include at least one course focused on obstetrical and paediatric content (CASN, 2017). At our university, nursing theory and clinical courses which include families and clients from pregnancy through childhood and adolescence are titled “young families”. In this context, young families clinical placements have traditionally focused on post-partum populations.

### Challenges obtaining young families clinical experiences

Clinical experiences are an essential component of nursing education for the consolidation and application of knowledge and skills learned in theory courses in practice. The clinical experience has evolved as nursing education has shifted from hospital-based diploma programs to contemporary baccalaureate education, moving from on-the-job, unit-specific training to preparation for the integration of a broad knowledge base into diverse work environments (CASN, 2015). Clinical education varies by program, but most include simulation and clinical experiences in acute care, chronic care, and community settings (CASN, 2015; Smith et al., 2013).

In Canada and internationally, all types of clinical learning experiences are becoming increasingly challenging to secure, particularly in hospitals (Franzese et al., 2020; Smith et al., 2013). This is due to factors, such as limited clinical sites and capacity, oversaturation of students, nursing shortages and understaffing (Franzese et al., 2020). These challenges worsen in paediatric and obstetrical care settings which are scarcer and have fewer patients than other hospital units (Díaz et al., 2021; Rusk & Murray, 2023; Sherman & Johnston-Merickel, 2021). Pohl et al. (2017) attribute the lack of paediatric clinical experiences to a shift in care to specialty tertiary centres, resulting in decreased patient census in acute paediatric units due to advances in care, as well as the prohibitive costs of paediatric care in small local hospitals. Additionally, tertiary care centres are often located in urban environments (Kubin et al., 2013) and have limited capacity to support learners (Rusk & Murray, 2023). In obstetrical settings, a gradual decline in the number of births is a continuing Canadian trend (Canadian Institute for Health Information, 2023). This, coupled with shorter postpartum hospital admissions and a shift in birthing preferences, is perpetuating the admission of off-service clients to obstetrics units and the movement to offer maternity and infant services in large tertiary hospitals only (Cobbett & Snelgrove-Clarke, 2016; Kozhimannil et al., 2018; Pohl et al., 2017). Overall, these challenges have led some nursing education programs to augment, or entirely replace, young families clinical placements with simulation to ensure students can meet required learning outcomes (Díaz et al., 2021; Guimond et al., 2019; Riley-Baker et al., 2020).

### Summer camps as innovative young families clinical experiences

In response to the challenges of securing meaningful clinical experiences, educational institutions have been pursuing innovative summer camp clinical experiences to meet outcomes for community, mental health, and elective paediatric clinical courses (Hensel et al., 2015; Saylor et al., 2018; Schick Makaroff et al., 2013; Smith et al., 2013). Predominantly in the United States and not within the young families context, summer camp has a proven track record as a suitable placement. Camp experiences are diverse, but typically involve nursing students living on-site for the duration of the camp (1–2 weeks) and interacting with campers as part of a multidisciplinary camp staff team. Most often, nursing students self-select camp as a clinical placement, attend camps for children and youth living with specific chronic conditions or disabilities and are supervised directly by a camp nurse (Hendrickx et al., 2020; Hensel et al., 2015; Moran et al., 2021; Nash, 1987; Saylor et al., 2018; Schick Makaroff et al., 2013; Sherman & Johnston-Merickel, 2021; South, 2010).

To date, few studies have evaluated the experiences and learning outcomes of nursing students attending overnight summer camp as a clinical experience. The evidence consistently highlights positive feedback from students, faculty, camp staff, campers, and campers’ families (Evans, 2017; Hendrickx et al., 2020; Moran et al., 2021; Nash, 1987; Saylor et al., 2018; Schick Makaroff et al., 2013; Sherman & Johnston-Merickel, 2021). The literature also provides evidence that summer camps offer a rich learning environment and opportunities to meet clinical course outcomes, however, outcomes were often specific to paediatric nursing or diabetes management learning objectives. For example, Hendrickx et al. (2020) found that students learned about diabetes, childhood conditions, and improved communication with children. They gained valuable experience in paediatric medical administration, encountering diverse patient situations. They successfully met the paediatric clinical course outcomes, including therapeutic communication, health promotion, nursing roles, developmentally appropriate assessment, and intervention, and applying critical thinking to patient care. Saylor et al. (2018) also highlighted the opportunities for nursing students to engage in holistic care, patient teaching, leadership, and teamwork during a camp clinical experience. These studies suggest that overnight summer camp can be a valuable clinical learning experience for undergraduate nursing students. However, most prior studies lacked rigorous methodology, including opinions and anecdotes rather than systematic data collection and analysis. Moreover, none of the studies examined the ability of students to meet course outcomes for a young families course, inclusive of both maternity and paediatrics; therefore, further research is needed.

### Student learning in clinical experiences

Once a clinical experience has been secured, additional considerations can impact a student's ability to learn and successfully meet course outcomes. This includes external factors such as negative attitudes from staff, feeling welcomed, scheduling, and complex or differing placement requirements of each clinical partner (Anokwuru & Daniels, 2021; Barnett et al., 2008; Cooper et al., 2015; Grobecker, 2016; Rusk & Murray, 2023). Learning is also influenced by internal factors, such as feeling a sense of belonging, student expectations, and perceived opportunities to apply theoretical knowledge in the practice setting (Cooper et al., 2015; Grobecker, 2016; Henderson et al., 2006; Kol & İnce, 2018). Specific to in-hospital young families clinical experiences, over time, students have voiced concerns, the most common being lack of interest, role strain, gender-based barriers, and difficulty coping with the sadness that can, at times, accompany this type of placement, which are consistent with the literature (DeVito, 2016; Mitra et al., 2018; Tzeng et al., 2009).

### Purpose and research question

**Figure 1.**
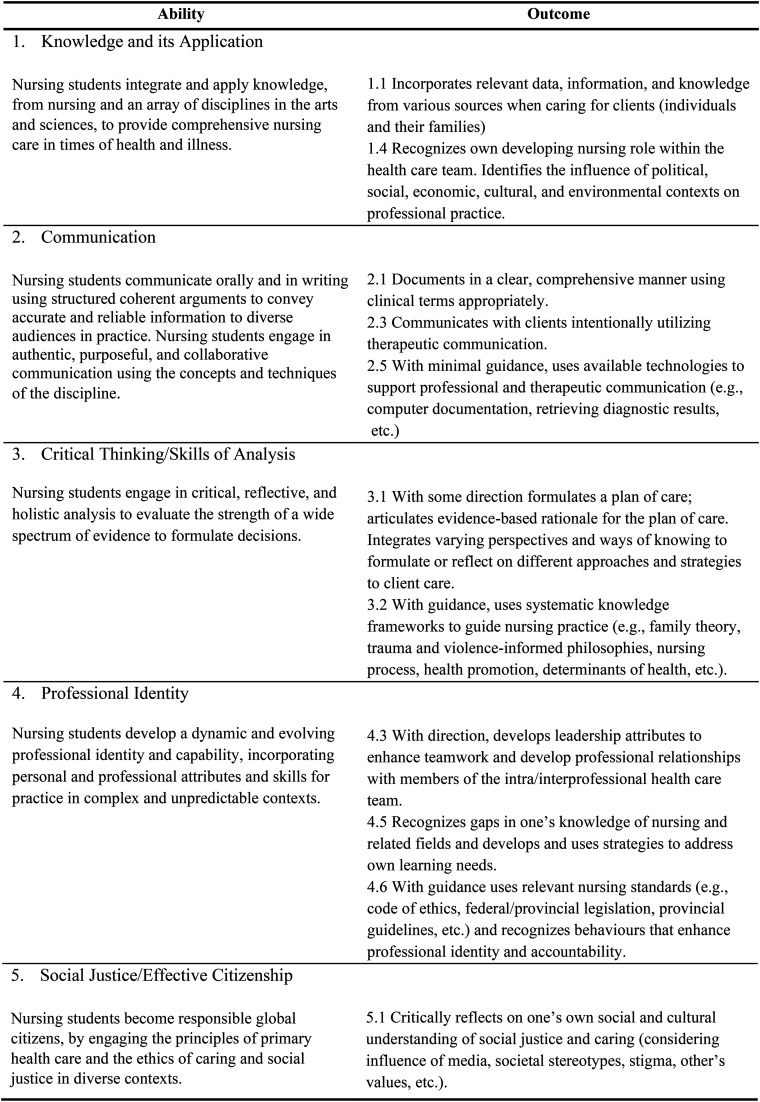
Course outcomes for young families clinical.

*Note.* Adapted from Faculty of Nursing (2021). NURS 2172: ASP clinical practicum course blueprint. Internal University of New Brunswick document: unpublished.

The main purpose of this study was to describe how students in an accelerated baccalaureate undergraduate nursing program met the learning outcomes of their young families clinical course using immersive summer camp experiences. This nursing program has a curriculum centered on five core abilities: 1. Knowledge and its application, 2. Communication, 3. Critical thinking/skills of analysis, 4. Professional identity, and 5. Social justice/effective citizenship, levelled and threaded throughout the program. These five program abilities are the basis for the learning outcomes of this young families clinical course (Figure 1). The learning outcomes are broad enough for students to meet clinical outcomes without being prescriptive about the setting and can apply to diverse young families populations (conception to age 18). The program abilities are tailored to fit each course within the nursing program, as each course has a different focus, and because students’ level of knowledge, skill, and ability increase as they progress. The overarching research question for this study was: *How were students able to meet the outcomes of a young family's clinical course using summer camps as clinical experiences?* A secondary purpose of this research was to describe the perceptions and experiences of students, camp administrators, camp nurses, and faculty involved in this placement experience by asking: *What are the perceptions and experiences of those involved in this clinical experience?*

### Study significance

The findings from this study provide knowledge about students’ ability to meet the learning outcomes of a young families clinical course using summer camp clinical placements. This study also highlights the use of summer camps as an innovative clinical learning experience given the growing limitations with traditional obstetrical and pediatric placements, providing valuable evidence for nurse educators interested in using summer camps to expand options for young families clinical experiences.

## Methods

### Research design and approach

Interpretive description methodology was used to identify themes and patterns related to the overarching research questions while acknowledging divergence arising from participants’ subjective perspectives (Thorne, 2016). Interpretive description acknowledges the researcher's foregrounded knowledge of the studied phenomenon and focuses on answering the posited question to produce tangibly practical and context-relevant implications for nursing application (Thorne, 2016). This methodology was selected for its flexible approach, allowing researchers to draw on their disciplinary logic and professional experiences to examine this phenomenon (Thorne, 2016).

### Positionality statement

The three female researchers (C.W., R.G., & E.R.) were faculty members in the nursing program the student participants were enrolled in. They held a decanal position, a clinical placement coordination position, and the lead educator position overseeing student participants in the associated clinical course. The team acknowledged their concurrent faculty roles may influence their assumptions and biases, potentially impacting the study. Researchers’ perspectives were shaped by literature and personal experiences as nurse educators, nursing students, and campers/camp staff. To address potential biases each researcher transparently acknowledged their perspectives (Thorne, 2016) and underwent a process of critical reflection (Dwyer & Buckle, 2009). Further, given the principal investigator's (PI) “insider” perspective as the clinical faculty member overseeing this course, peer check-ins with co-authors were conducted as a form of disclosure and team reflexivity to confirm shared agreement and understanding throughout all phases of this study (Dwyer & Buckle, 2009).

### Setting and context

The summer camp clinical experience took place in the summer of 2022, in term three (May-July) of a six-term accelerated Bachelor of Nursing (BN) program. Students at our university take both a theory and clinical course focused on young families. The purpose of the young families clinical course is to:

Provide students, in partnership with clients, opportunities to explore family processes, and develop and implement strategies to support health obstetrics practice behaviours of young families. This integrative practice experience will provide students with an opportunity to apply concepts of growth and development, family centered care, health promotion, and communication with clients, individuals, and families (Faculty of Nursing, 2021).

Historically, these courses have placed a strong emphasis on perinatal care and maternity/obstetrical care, with less focus on children and adolescents. In the past, clinical placements were mainly completed within inpatient obstetrics due to small inpatient pediatric units with low census and off-service adult admissions. In this novel approach, summer camps attended by the students were located within Atlantic Canada, ranging in length from six to ten days, were staffed by counsellors and administrators, and had at least one Registered Nurse (RN) on site. Students were assigned to four different camps and attended in pairs, for support and debriefing on-site. Camp staff hosted campers with lived experience of various conditions including cancer, diabetes, physical and developmental challenges, and campers experiencing socioeconomic challenges.

Nursing students self-selected to attend immersive summer camp clinical placements. This meant they stayed on site for the camp, participated in all camp activities, and learned in a camper-centered care model. This supported the philosophy of the nursing program and targeted outcomes of the young families clinical course.

**Figure 2.**
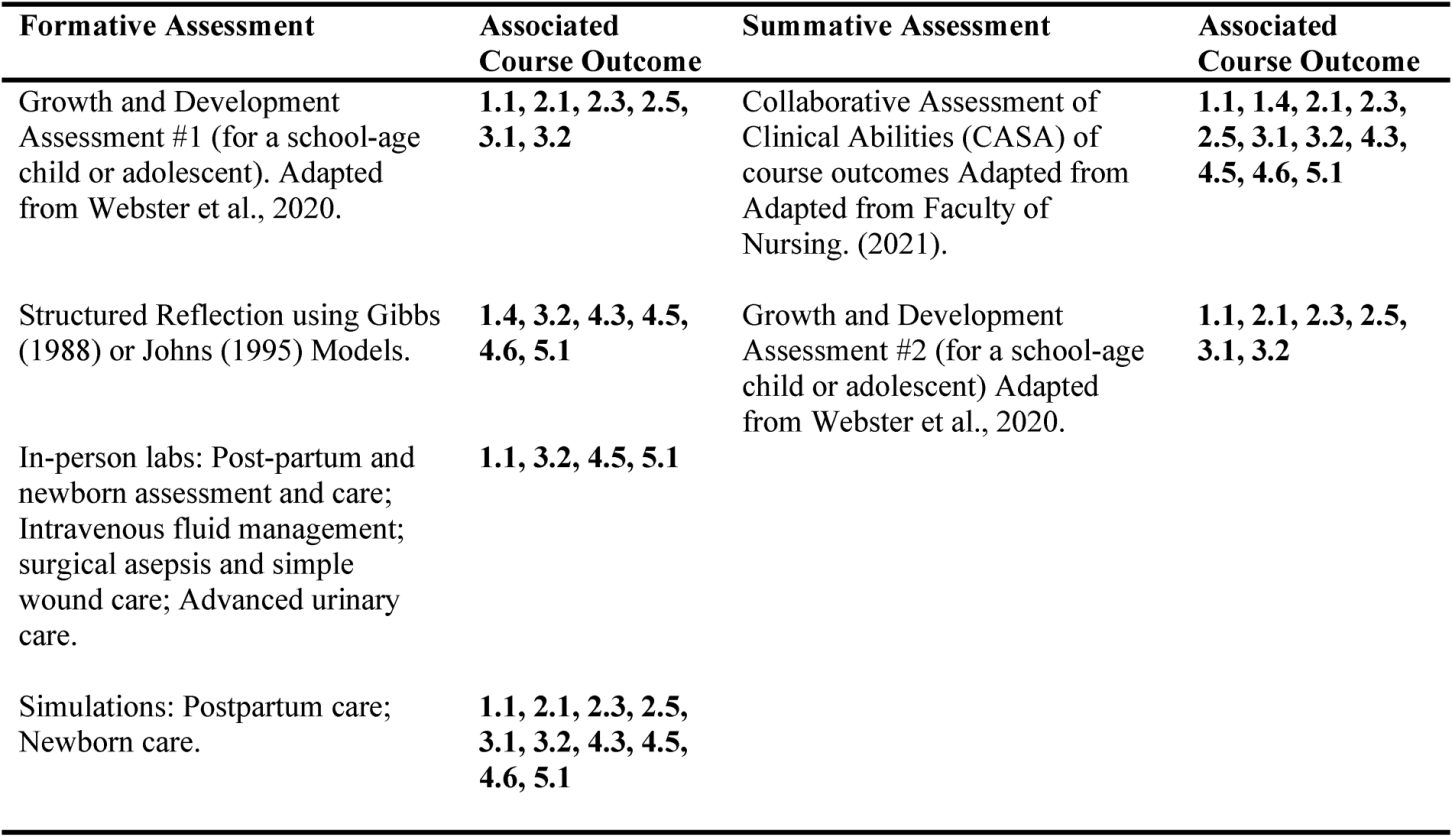
Assessment methods for young families clinical and associated outcomes.

*Note.* Adapted from Adapted from Faculty of Nursing (2021). NURS 2172: ASP clinical practicum course blueprint. Internal University of New Brunswick document: unpublished.

The clinical educator overseeing these placements (C.W.) was responsible for connecting with camp directors and nursing staff, explaining course expectations to staff working with the nursing students, clinical debriefing with students (virtually and in person), and evaluating formative and summative student assignments including two growth and development assessments for a school-aged and adolescent child, a structured reflection, and collaborative assessment of student abilities (CASA). All formative and summative assignments aligned with course abilities and outcomes (Figure 2). The clinical educator communicated with students by phone and travelled to sites halfway through each placement.

To ensure students selecting a camp experience were provided with an opportunity to practice obstetric skills, students completed in-person skills labs on newborn and postpartum assessment and virtual simulation on postpartum care. As students were unable to attend concurrent theory classes during their week at camp arrangements were made to accommodate students (e.g., recording lectures, and no tests scheduled during camp weeks).

### Participants and sampling

Participants were purposively recruited between October and November 2022 to explore the perspectives of individuals involved in the camp clinical experience in different roles. Those eligible for participation included students who participated in summer camp clinical experiences (N = 8), camp directors from summer camp placements (N = 4), camp nurses who either acted in an advisory capacity or worked directly with students (N = 5), and the clinical educator responsible for placement oversight (N = 1).

Camp directors and nursing staff received an email invitation from the PI. Students received an email invitation after receiving grades for the associated course. Interested parties contacted the PI directly. One-hour semi-structured interviews were conducted virtually by the PI with participating camp administrators including camp directors (n = 3) and a camp nurse in an advisory role (n = 1). Interviews varied between 12 and 65 min. A two-hour in-person focus group was conducted with student participants on campus (n = 4). The clinical educator overseeing this placement, who was also the PI for this study (n = 1) was interviewed by another member of the research team (R.G.). This was deliberate, as this person brought an important perspective to the research because of the embedded nature of the role and their subsequent experience in a professional context.

### Data collection procedures

All interviews were conducted virtually between the PI and individual participants via Microsoft Teams. Interviews were recorded and auto transcribed, and interviewer field notes were handwritten. After each interview the responsible researcher reviewed and corrected the transcript, deleting recordings upon verification. The in-person focus group including only the researcher and nursing student participants was audio recorded, and transcribed verbatim by the interviewer, with recordings deleted post-transcription. Pseudonyms were assigned to interviewees for transcript identification. Details potentially identifying participants were removed, and anonymized transcripts were securely uploaded to a private, password-protected Microsoft SharePoint site for exclusive access by the researchers.

### Data analysis techniques

Transcripts were analyzed using interpretive description methodology (Thorne, 2016). After data collection, all three members of the research team reviewed transcripts individually, using inductive coding and constant comparative analysis (Thorne, 2016) to develop themes and sub-themes which responded to the research questions. The research team then met to discuss and reach a consensus on themes and sub-themes, during which time a collaborative process was followed, integrating the perspectives of each author, equally.

#### Rigour

Methodological rigour was preserved using criteria outlined by Thorne (2016) and Jackson et al. (2022) to the best of the authors’ abilities, which includes: epistemological integrity, including methodological congruence (e.g., co-author collaboration regarding methodological understanding) and peer review (e.g., findings were presented at a research conference). Representative credibility, inclusive of appropriate sampling (e.g., combining results from various roles and perspectives), comprehensive use of participants’ direct quotations, and the amalgamation of participant and researcher perspectives to support findings. Analytic logic using an audit trail (e.g., author notes reflecting on data collection and analysis). Lastly, interpretive authority was established through the verification of interview transcription and ongoing and frequent discussion around data analysis and interpretation.

### Ethical considerations

Research Ethics Board approval (REB 2022-145) was obtained from the affiliated Atlantic Canadian University, and written consent from participants was obtained before data collection. The PI, who was also the clinical educator, avoided contacting participants until the course was complete. Additionally, this educator only teaches in the first year of the program, therefore, as students were transitioning to another year of the program following this course, the PI would not have any formal interaction with student participants after this time point. Participants received a $50 gift card as a thank-you.

## Results

### Participant characteristics

Nine participants, four students, four camp administrators, and one clinical educator participated in this research, of whom 67% identified as female, 22% identified as male, and 11% identified as non-binary. Forty-five percent were 26 to 30 years old, 33% were over age 40. Most participants (77%) previously attended summer camp, many in a camp counsellor role. All participants had completed some post-secondary education. A detailed description of the study sample is presented in Table 1.

**Table 1.** Participant characteristics.

Characteristic	*n*	*%*
*Gender*		
Female	6	67
Male	2	22
Non-Binary	1	11
*Age*		
20–25	1	11
26–30	4	45
30–35	0	0
36–40	1	11
>40	3	33
*Previously attended summer camp*		
Yes	7	77
No	2	23
*Capacity attended camp previously* ^a^		
Camper	4	
Counsellor	7	
Nursing Staff	1	
Administration	4	
Other	1	
*Post-secondary educational background* ^b^		
Arts	1	
Nursing	2	
Science	6	
Other (Education, Law)	2	

*Note*. *n *= 9.

^a^ Some participants have previously attended camp in multiple roles.

^b^ Some participants have multiple degree

### Emergent themes

#### Students’ ability to meet course outcomes

To answer the focal research question of this study, themes and subthemes originating from interview and focus group data were organized using the *a priori* program abilities from which the course-specific outcomes are derived (Figure 1). This was done by pairing emergent *a posteriori* themes and subthemes with the most applicable program abilities (Table 2). The results are summarized below.

**Table 2.** Ability to meet course outcomes.

Ability	Theme	Subtheme	Example Quotes
1. Knowledge and its Application	Unique environment	Unique learning experience	“It's all about experiential education and learning. That's what we do. Camp is a tool to facilitate these things and build community. And we build community on a multitude of different levels from the camper population to the families population.” (Participant B) “I love it when I get an opportunity to show students that nursing is not all hospital nursing … you use so much more nursing than you think you’ll use [at camp].” (Participant C) “it was a cool opportunity to see, I guess like what nurses can do outside the hospital.” (Participant F) “I liked that it was like, like, living the experience of it. Because it was cool to just like be there and be like constantly working … for the 10 days we were there.” (Participant E)
	Application	Nursing skills	“I really like the environment of like just like fun that the kids having at all the activities. We got to do more of, like, a kind of first aid cuts and scrapes and sprains type of role, which was a lot of a lot of fun to get to learn more of those kinds of assessment skills and practice those skills.” (Participant H) “So many meds! … I feel like we learned more … Just the administration and stuff that has to happen legal-wise.” (Participant E) “on the first day for our camp like the parents had to sit down with the nurses. Here's the medications the nurses were writing out MARs and then, like, making sure they went over every single medication before the like the parents or guardians even left.” (Participant G) “it's one thing to learn about developmental theories than it is to, actually like apply them.” (Participant F) “They got to really work on their assessments of the paediatric population, which is not a population that they get to see a lot in clinical practice, even if we were to do like a paediatric intense block of clinical, I really don't think they get exposed to this many kids, and they got to see them in varying levels of health.” (Participant I)
2. Communication	Application	Therapeutic communication	“there's a lot of communication that happens a lot of learning how to speak in lay terms, especially with adolescents … You talk to teenagers using their language and you would do your very best to be approachable.” (Participant C) “They also got to do a lot of talk therapy and getting to know clients and interacting with them on their level. It's hard talking to adolescents and kids if you're not used to it, and we can't talk to them the same way that we talked to our colleagues. So that in itself is a very meaningful and useful piece of learning.” (Participant I) “It was fun, but at the same time we got to make, like, build connections with the kids.” (Participant G)
		Documentation	“we filled out so many DARP notes! I probably did like 10 DARP notes a day because kids every time we give them anything or for anything we had to fill one out.” (Participant E)
3. Critical thinking/Skills of Analysis	Praxis	Clinical judgment	“sometimes you look at the medication and they're contradicting each other. So, then he would get on the call at that point and talk to pharmacists and saying this seems a bit odd. And they would find certain things. And I think that type of exposure, that type of process and that type of, well, that that type of thought process is really important.” (Participant B) “Being able to pivot quickly … somebody suddenly has an asthma attack or anaphylaxis or something like that, and although these emergencies don't happen often, they are unpredictable. So, there is a lot to be said about the ability to be able to grab your trauma kit, respond, and without all the support like traditional nursing … so, there is a lot of learning.” (Participant C)
	Building confidence	Growth	“Student nurses learn from the other nurses that are there, and I think people learn about themselves when they leave camp.” (Participant A) “It's a great place to build your confidence because once you respond to your first couple of emergencies you realize, “I can do this”, you know, “I'm capable”. (Participant C) “Sometimes there was negativity and … an unwillingness to completely involve themselves in what was going on, they had distinct feelings about how they were going to meet the objectives because ‘we were nursing’, and ‘we were only doing nursing things’, and as we all know, there is a lot more to nursing than just giving out medications and assessing wounds. There is the social part of things, and the helping part of things, and it doesn't mean physically doing something always. And I think it took some people a little bit longer than others to make that connection. And that camp is a perfect way to make connections and help people in a non-physical way.” (Participant I)
4. Professional Identity	Intra/interprofessional collaboration	Developing Professional Relationships	“it's not new for us to have like [students] it just you kind of forget who's medical and dieticians and they kind of all blended in at one point because we there's so many of them! But I don't know, it's just I think it's part of camp everybody's learning …” (Participant D)
		Teamwork	“They got to work with multidisciplinary teams at lot of the camps to help manage the different needs of the campers. They got to see how these teams really interact in a non-acute setting, which was incredible.” (Participant I) “you kind of forgot they were students versus the regular medical staff at that point! Everybody just worked hands on.” (Participant D)
		Networking	“I also think we build community on the medical side of things as well. Like, I would hope these students also now have maybe are still young enough that they, but if they come back, they’ve developed a relationship like with our [physicians] … there's so much, other resources that if they're later in life they could probably just connect with them and say: “Hey! I've seen this case. Could you just give me some ideas?” (Participant B)
5. Social Justice/Effective Citizenship	Campers are more than their diagnosis	Decreasing stigma	“there's this huge stigma and fear around it [caring for people with disabilities] and if you have any experience or exposure, it eliminates that kind of fear on both parts.” (Participant A) “My camp was all kids who weren't neurotypical … what was really special about it is you could tell that none of the kids felt like they were being judged at all or anything like that. Like, all the kids could just be themselves and like it was just really fun to see.” (Participant G) “it was interesting, I guess, to like partake in the activities because like after having like read some of their files and like seeing like the difficulties they had like some of them like were only a few years old when they had cancer diagnosed. So like seeing them, as you know, like 8 to 10 year olds or older, like having fun and like being normal kids like, beyond their cancer diagnosis and remission like was really heartwarming, and I think like the Canadian Cancer Society nurse that was there, like really summarized it well, it's like it's a good spot to realize that, like beyond illness like there's still people and they still continue to like, have fun, and have lives.” (Participant F)
		Reflecting on personal values	“humanizing the chronic illness or the disease in a certain sense and seeing that piece. I think then it also allows a lot of opportunity to learn on, again, chronic illness.” (Participant B) “I think it's great … it's very helpful how to break down barriers and maybe preconceived notions about [chronic illness].” (Participant D) “It was different than, like, sick kids that you'd see in the hospital.” (Participant E)
	Acknowledging societal stereotypes	HCP are people too	“The goal for us is also about kids seeing medical professionals in clinical environment as humans, as people that could have fun … I truly believe that there's a bit of an empathy that's built into that program for students.” (Participant B) “I'm a big advocate of … camp nurses being out in programs, up on the climbing wall, down at the waterfront, giving out sunscreen because oftentimes for our population … kids who are economically disadvantaged … they don't have regular health care providers, so they're experienced with healthcare tends to be emerge [ED], and it tends to be incredibly negative. So, just being able to have them see nurses as not scary people; people they can ask questions to, that are approachable. I think is huge. It's all about trust and trusting the healthcare system.” (Participant C)
	Social justice in diverse contexts	Expanding awareness	“there's a girl that came into the little nursing station that we had and I remember her telling us that she felt that camp was very peaceful because that she knew that that camp there was food on the table. She didn't have to worry about it, so that really put everything into perspective knowing that it's like a low-income camp and that you know a lot of people are probably feeling like that for her to just say that to us was very impactful.” (Participant H) “Social justice and citizenship … the camp ones were incredible. What they learned, what they pulled from it, how they were able to see themselves as citizens, how they were able to help and apply the social determinants of health to the population they were with, they were incredibly sad, but they also made them uplifting because they were describing the living conditions or situations from which the campers are coming from, and how camp is making a difference in these campers lives, more depth than even the community health course; I’ve never read outcomes that have been that deep and meaningful.” (Participant I)

##### Knowledge and its application

Participants spoke about the requirement to adapt their practice to meet client needs in the unique camp setting, which responded to course outcome 1.4*.* Further, participants spoke about applying previously learned skills such as health assessment and medication administration, and the application of developmental theories in this context, which related to course outcome 1.1.

##### Communication

Participants shared that there was ample opportunity to practice documentation which connected to course outcome 2.1. Participants also shared that refining communication skills to build connections with adolescent clients was a key component of the camp experience which related to course outcome 2.3.

##### Critical thinking/skills of analysis

Participants shared examples of how critical thinking skills were developed during this placement. Additionally, while care planning was not part of the camp experience, data indicated that nuanced informal care planning was taking place with the relational elements of this experience and as higher acuity situations presented. These examples fit with course outcome 3.1.

##### Professional identity

Participants shared information about personal growth, opportunities to develop relationships with other health professionals, practice teamwork, and network which supported course outcome 4.3. Multiple references were made to growth through exposure to new experiences which concurred with course outcome 4.5. Beyond this, reference was made to learners recognizing their obligations in this unique setting, which connected to course outcome 4.6.

##### Social justice/effective citizenship

This program ability had the richest data, which was unsurprising given the nature of the partnering summer camps, including camps for adolescents living with chronic health issues and adolescents experiencing social and/or economic disparities. Participants described opportunities to reflect on their own and others’ values, expand their awareness, and learn about practicing person-centered care, relating to course outcome 5.1.

Finally, participants made comments which supported the sentiment that students were able to meet course outcomes, such as Participant I, who stated, “they were absolutely able to meet the outcomes and, in some cases … were able to meet them better than they were in the obstetrical experience.” Of note, while students met course outcomes overall, there was limited evidence from the triangulation of multiple sources to support two course outcomes, 2.5 and 3.2, based on collected data. However, the clinical educator overseeing this experience shared in their interview that each camp used technology for documentation (2.5), and students used knowledge frameworks in the required formative assignments (3.2), providing evidence that students met these outcomes.

#### Participant's perceptions and experiences

The research team collated applicable data to develop themes and subthemes (Table 3) to answer the secondary research question: *What are the perceptions and experiences of those involved in this clinical experience?*

**Table 3.** Participant perceptions and experiences of placement.

Theme	Subtheme	Example Quotes
**Learning environment**		
Exceeded expectations	Helpful	“students were really a huge benefit to us from a people power perspective because it wouldn't have been feasible for us to hire all these, let's say, have all RNs.” (Participant B) “I know it was a learning experience for them, but it was extra hands-on for us and they were really eager to help out and cooperate and learn. So yeah, it was nice to have extra people.” (Participant D)
	Positive experience	“… they were a lot more help than we thought they'd be. And that was not in a bad way, they were just so eager and enthusiastic and wanted to do everything … we might have set this up for failure because by sending us such great candidates, it set the bar really high!” (Participant C) “we had a great experience.” (Participant D) “They want students who want to be there. And just the insightfulness, the acceptance, the openness, and willingness to learn. They expected it, but not to the level that our students gave them their involvement. They were willing to do anything that was asked, and they just thrived in that environment, and they were just so happy to see it. They loved having extra sets of hands to help with things … they just really enjoyed their enthusiasm and their energy.” (Participant I) “I just love camp environment so it's nice to be back there and I thought it was interesting to have, like, a more of a role where we had pretty big responsibility.” (Participant G) “The food was really good, like, really good. I ate a lot of food!” (Participant E)
	Ongoing opportunity	“… more of these types of partnerships are very beneficial for us.” (Participant B) “We're happy to have more students … because it was great.” (Participant C) “… look at our camp experiences for students … let's just make sure ABC we get for them because that is what I would look for in a clinical experience.” (Participant C) “I just hope it keeps going. The partnership keeps going because I think it's great … it's very helpful how to break down barriers and maybe preconceived notions about [chronic illness].” (Participant D) “One camp in particular had never had students before, and they were also the strongest advocates for having them return and they would like them to go to multiple camps … Another camp had not previously had nursing students in the capacity of doing a required clinical. And again, they really enjoyed it.” (Participant I)
Opportunities for improvement	Role confusion	“I thought my camp experience didn't have enough structure and so, like my partner that I was with for the experience, like him and I often like would help out with a few activities and then, like, there was nobody to tell us what to do or like there really wasn't anything for us to do so like we just did nothing or like we would go off and do our own thing. So, there was like definitely periods where I didn't feel like I was learning anything. Some more structure I guess would have been nice.” (Participant F) “So I found like making connections with the other staff members was really hard, because we were only there for one session … we didn't really know the schedule that way of what the kids were doing … There's definitely that disconnect between the staff members and like ourselves.” (Participant E) “even like probably the 4th or 5th day. Like there were people who just didn't know why we were there because they didn't know that we were like there as nursing students. We were just like new people who showed up.” (Participant F)
	Educator interaction	“Is it beneficial to have a person like yourself that teaches nurses nursing, or others that are onsite with the students to do some? I don't know if we're missing any gaps … you might be able to connect other learning pieces.” (Participant B)
	Camp nurses	“It also would have been great if I had more access to the nurses that were actually working at the camps … and I understand a lot of that is because a lot of the time they don't really know who the camp nurse is going to be or who's going to be helping the students out while they're there. I've learned that. Personnel and staffing is a huge issue, even at summer camp … I did find that kind of frustrating – not being able to talk directly with the nurses, and sometimes even when I did have their contact information then they didn't respond anyway. But knowing that I did put it out there for them so that if they need to contact me, I was very open and willing to share with them at or assist them in any way.” (Participant I) “Some of the nurses said that they didn't really understand why the students were there. They didn't know what their role was. And I think that contributed to how the students felt at the camp. They didn't necessarily feel included, and I don't know if it's just an individual thing or if it was more.” (Participant I)
Right fit	Right personality	“I certainly have the personality … for camp like I'm really loud. I love to sing, I love to be silly, I love to dress up. And so, it was like a natural fit for me to be in a camp setting.” (Participant A) “… It takes a certain personality, certain pieces. So, I think the thing about this is not everyone, not everyone is geared or maybe, umm, flexible enough to be in the camp environment.” (Participant B) “I think it requires a certain amount of preparedness and reality check when we have our interviews. We often talk about the fact that this is not a clinical setting. This is not a hospital setting and if you've only worked in those settings, you've got to be recognizing that this is not it. So, I think there's been that approach that some say that they're ready, but when they get there, they're like, you can tell that they've … had no experience with children and they've had nothing but clinical experience, so their approach is a little bit more rigid … so I think that is a bit of an ongoing challenge.” (Participant B) “We've seen people come in on 1st day in and leave because they're like: “Nope, can't do it.” And so, I think there's a few things, like, that … I've gotten better over my career of acknowledging that and making sure people are ready and understand exactly what that means.” (Participant B) “if they really don't want to be there, … that just makes a negative experience for everybody else.” (Participant C) “I think that because of their acute experiences in the past, they felt that they had to stay with the nurses and just do nursing things. But as time went on and they realized that they could kind of relax a little bit from that, it got better, and they ended up enjoying themselves. So that was a little bit of a challenge initially.” (Participant I) “Yes, I saw, in some cases, reactions from students that I wouldn't have necessarily expected to certain things. I don't know if it was because of lack of sleep or being at camp and interacting and being ‘on’ like 18 h a day.” (Participant I)
**Learning outcomes**		
Positive alternative	Relief of not having to complete an obstetrical experience	“a lot of students don't enjoy obstetrics … so it is nice to be able to offer them something a little bit different that still meets the objectives of the course, but also makes them more comfortable.” (Participant I) “I'm very happy I never had to go the to maternity floor! I have no interest in it.” (Participant E) “identifying as a male like, it is a challenge in itself to kind of interject myself in the maternity unit.” (Participant F)
Concern	Missing out	“they're limited in what they're allowed to do when and what they need to be supervised doing.” (Participant D) “one thing that I found hard with going to summer camp was that we use clinical a lot to like solidify what we learned in class. And in class we're learning about babies and pregnancy and so I found it harder for the exam to not have that like, practice, and I'm a little worried about the NCLEX because I still feel that that's my like weak spot. But I did really enjoy the experience, just like for putting it together with class stuff. It helped with the developmental stuff, but the baby stuff, I'm like, I know nothing about this.” (Participant H) “I wish that we could have done maternity too. It was a hard choice for me because I really wanted to go to camp, but I also really wanted to see what maternity was like.” (Participant G) “I think like the option, even just do like 3 shifts or like 2 shifts [on OBS] or like maybe would be like a good compromise.” (Participant F)
Benefits and challenges to meeting outcomes	Making the Connection	“I really liked reading their CASAs. They were really, really well done. Providing me with extraordinary examples of how they were meeting the outcomes, and I found this incredibly rewarding, especially because a lot of the students had initially voiced concern about their abilities to meet the stated outcomes for the course. And they met them all incredibly well.” (Participant I) “The connections that the students were making with the campers on such personal levels and on such helping and caring levels, the activities that they were able to engage with the campers were so much more than just casual social interactions. They were actually involved in caring and helping relationships. They were making campers’ lives better. They were enabling them to be themselves. They were enabling them to enjoy this week of their lives, because a lot of campers that come to the camps that we sent the students to have a lot of things going on in their life, whether it be chronic illness, living in poverty – that just it runs the gamut of the social of the determinants of health. So, I think it was a really great place for the students to see these in action as well as be able to help in a really meaningful way.” (Participant I) “I stretched my CASA pretty good, but again, I think that again, is just the camp not being on the same page. I think I would have met the outcomes if I had gone to any other week like, very easily.” (Participant F)

**Figure 3.**
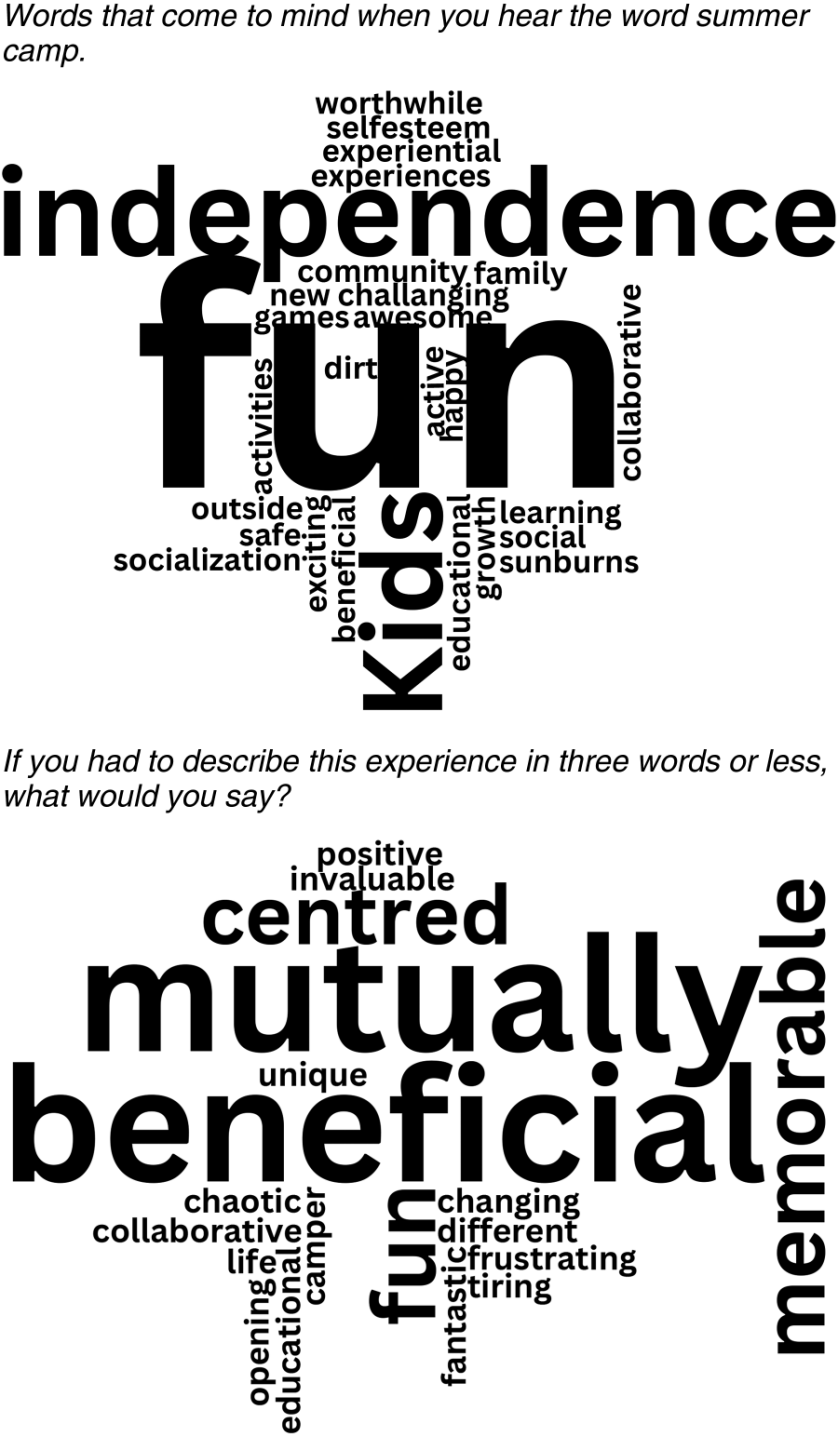
Words that come to mind when you hear the word summer camp. If you had to describe this experience in three words or less, what would you say?.

Two ‘word clouds’ were generated using NVivo to visually represent participants’ perceptions of summer camp (Figure 3). The first represents responses to the question “What words come to mind when you think of summer camp?” The second aggregates participants’ three-word descriptions of their summer camp experience. Word size corresponds to response frequency.

Overall, participants had positive views, with the first word cloud depicting a fun environment emphasizing independence, socialization, collaboration, growth, and learning. The second word cloud captures participants’ optimistic and constructive descriptions, highlighting mutually beneficial, memorable, fun, challenging, chaotic, frustrating, and tiring aspects of their experiences.

Globally, the data showed that these experiences exceeded expectations. Students were seen as eager to learn, positive, and helpful. All participants spoke about their interest in continuing, and in some cases expanding placement partnerships. The data emphasized the significance of a “right fit” or personality match for student nurses in their clinical experience. Positive fit traits included being outgoing, flexible, and open-minded, while negative fit traits encompassed disinterest in placement, close-mindedness, and rigidity. Given this, providing students with the option to self-select this placement was seen as beneficial. Lastly, this placement was seen as a positive alternative to inpatient obstetrics which was the other placement option offered for this clinical course.

Data also exposed opportunities for improvement. This ranged from individual experiences, such as one participant indicating that they had difficulty making connections with camp staff, and another sharing they felt there was not enough structure or support on-site during the placement, to broader issues such as challenges with camp RNs’ ability to adequately support learners on site. These included the clinical educator having limited access to RNs and the need for clearer role expectations for both camp RNs and nursing students. Lastly, some concern was noted around learners feeling they were ‘missing out’ on learning opportunities they may have experienced in in-patient obstetrics.

## Discussion

Students in an Atlantic Canadian BN program successfully met the outcomes of their young families clinical course using overnight summer camps for children and youth as clinical placements. The experience was perceived positively by study participants, aligning with past research on the benefits of such placements. To our knowledge, this is the first qualitative study examining summer camp as a clinical experience to meet the outcomes of a young families clinical course, thus our findings make a novel contribution to the literature.

The findings of the current research are consistent with those from past studies. Previous research has noted the benefits of summer camp placements for nursing students including increased confidence, understanding of disease management outside hospital settings, and opportunities to practice and develop a variety of nursing skills including medication administration, leadership, teamwork, and interprofessional collaboration (Hendrickx et al., 2020; Moran et al., 2021; Nash, 1987; Sherman & Johnston-Merickel, 2021). Similarly, we found this placement provided nursing students with the opportunity to build positive therapeutic relationships with children and youth living with various chronic conditions or social disadvantages, further develop and apply their nursing knowledge, skills, and critical thinking abilities, enact leadership, and work as part of an interdisciplinary team.

Unique to our study was examining summer camp as a placement option for a required clinical course focusing on young families inclusive of obstetrical and paediatric nursing care. Previous studies have focused almost exclusively on paediatric-specific nursing courses, many of which were elective courses (e.g., Nash, 1987; Saylor et al., 2018). The outcomes for our young families course align with the current entry-level competencies (ELCs) for RNs in New Brunswick which take a lifespan approach (i.e., there are no ELCs specific to paediatric or obstetrical nursing) and are flexible enough to apply to a variety of clinical settings (Nurses Association of New Brunswick, 2019). In a previous study, Evans (2017) mapped opportunities for fourth-year nursing students to meet the 2014 ELCs for RNs in Ontario through an overnight summer camp clinical experience, from the perspective of RNs who had been camp nurses. The current research corroborates these findings using the first-hand experiences of nursing students, camp administrators, camp nurses and nurse educators, demonstrating that young families course outcomes can be met at summer camp. This adds to the evidence that overnight summer camp is a valuable clinical placement option for young families clinical courses, particularly within the context of increasing nursing student enrolment and limited access to traditional obstetrics and paediatric clinical placements.

Findings from the present study related to participants’ perceptions and experiences are in line with those reported in the literature, in that the camp experience is generally positive for students (Hendrickx et al., 2020; Hensel et al., 2015; Nash, 1987; Saylor et al., 2018; Schick Makaroff et al., 2013; Sherman & Johnston-Merickel, 2021; South, 2010). Consistent with previous studies, participants in our study reported that camp was a unique context for learning that was eye-opening in terms of understanding the lived experience of children and youth with ongoing health and social challenges as well as broadening their view of nursing and health.

The results also indicated that nursing students appreciate having options for their young families clinical placement. Some students expressed no interest in obstetrics and were relieved to have an alternative option, while others had an interest in paediatric and adolecent populations, preferring the summer camp option. Although not reported directly in our research, there is evidence that students who identify as male may feel awkward or unwelcome in obstetrical clinical placements (DeVito, 2016; Mitra et al., 2018). For these reasons, having options within young families clinical courses is important. Some students would have liked to have had placements with both obstetrical and paediatric populations, and having selected the summer camp option, they felt they might be missing out. While in-person obstetrical experiences may not be possible for students choosing the summer camp option, learning can be supplemented with skills labs and simulation as needed (Pohl et al., 2017; Saylor et al., 2018).

### Study limitations

It is possible that the participants who did not partake in the study had different perspectives about the experience that we were unable to capture. In addition, RNs working directly with learners at camps did not participate in this study, despite invitation. This may be because of the temporary nature of the camp nurse role at most camps. Lastly, the inclusion of the PI as an “insider” during this research process may be seen as a study limitation. Conversely, this may be seen as a strength, depending on one's perspective. Ultimately neither approach assures the trustworthiness of the research (Dwyer & Buckle, 2009).

### Implications for nursing education

The findings of this research add to the evidence supporting the use of overnight summer camps for children and youth as a young families clinical placement option for nursing students. Practical recommendations emerged for nurse educators considering this model. For example, clear roles and expectations should be established from planning to conclusion. Nursing students need thorough orientation to both the clinical course and camp. As camp nurses may be transient, connecting during their orientation allows the clinical educator to explain the placement, provide materials, and address inquiries directly. Providing schedules beforehand, clarifying activities with camp administrators, and introducing students to camp staff are crucial steps. Effective communication with camp staff is vital due to potential variations in student nurse roles.

### Suggestions for future research

Consistent with interpretive description, this study focused on answering specific research questions. However, data captured for this study led researchers to consider future research related to summer camps including camp work culture, training and support for camp staff, and opportunities to explore interprofessional education and engagement at camps in which other healthcare professionals/students are present. Future research may also include similar studies which consider the use of summer camps as clinical experiences for mental health or community clinical courses in a Canadian context.

## Conclusion

Students who participated in summer camp as a clinical placement for a young families clinical course were able to successfully meet the course outcomes. The experiences and perceptions of those involved in these experiences were overwhelmingly positive. However, opportunities for improvement were also noted. Study findings may inform nursing education curriculum, in the context of using alternative clinical experiences such as summer camps for courses involving young families content.

## Supplemental Material

sj-docx-1-cjn-10.1177_08445621241288489 - Supplemental material for Summer Camp Clinical Placements in Young Families Nursing: An Interpretive Description Study Supplemental material, sj-docx-1-cjn-10.1177_08445621241288489 for Summer Camp Clinical Placements in Young Families Nursing: An Interpretive Description Study by Claire Williams, Renée Gordon and Emily Richard in Canadian Journal of Nursing Research
